# MiR‐29b regulates Th2 cell differentiation in asthma by targeting inducible B7‐H3 and STAT3

**DOI:** 10.1002/clt2.12114

**Published:** 2022-01-18

**Authors:** Wenjing Gu, Gang Li, Weili Zhang, Xinxing Zhang, Yanyu He, Li Huang, Yongdong Yan, Wei Ji, Chuangli Hao, Zhengrong Chen

**Affiliations:** ^1^ Department of Respiratory Medicine Children's Hospital of Soochow University Suzhou China; ^2^ Institute of Pediatric Research Children's Hospital of Soochow University Suzhou China

**Keywords:** asthma, B7‐H3, macrophages, miR‐29b, STAT3, Th2 cell

## Abstract

**Background:**

MicroRNAs play an important role in T cell responses. However, how microRNAs regulate Th cells in asthma remains poorly defined.

**Objective:**

In this study, we investigated the mechanism and pathways of miR‐29b regulating Th cells in asthma, in order to find new targets for asthma.

**Methods:**

We detected miR‐29b, B7‐H3 and STAT3 in the peripheral blood of children with asthma, explored the relationship between these molecules and their effects on T cells through in vitro cell culture, and verified it by animal model.

**Results:**

MiR‐29b levels were decreased in the peripheral blood mononuclear cells from children with asthma. Vitro studies found that the expression of miR‐29b in macrophages was decreased and the expression of B7‐H3 and STAT3 was increased after house dust mite (HDM) stimulation. After down‐regulation of miR‐29b in macrophages, the expressions of B7‐H3 and STAT3 in macrophages were increased and T cells differentiate into Th2 cells. After the addition of B7‐H3 or STAT3 antibodies, the differentiation of naive T cells into Th2 cells was reduced. In OVA induced mice asthmatic model, after the up‐regulation of miR‐29b in lung, the expression of B7‐H3 and STAT3 decreased in the lung tissues of mice, and the expression of Th2 cells and type II cytokine decreased simultaneously. The pathological changes of lung tissues were also alleviated.

**Conclusion:**

The expression of miR‐29b is decreased in asthmatic children. MiR‐29b can inhibit Th2 cell differentiation by inhibiting B7‐H3 and STAT3 pathways at the same time, and reduce asthmatic immune inflammation.

## 1 INTRODUCTION

Asthma is defined by the history of respiratory symptoms such as wheezing, shortness of breath, chest tightness and cough that vary over time and in intensity, together with variable expiratory airflow limitation. Asthma affects an estimated 300 million individuals worldwide and is the most frequent non‐infectious disease in children. It is a serious global health problem affecting all age groups, with increasing prevalence in many developing countries, rising treatment costs, and a rising burden for patients and the community.^1^

Asthma is characterized by increased airway inflammation and remodeling of airways and dysregulated type 2 immune responses. This results from sustained exposure to aeroallergens, which lead to elevated concentrations of type 2 cytokines including IL‐4, IL‐5 and IL‐13, alongside allergen‐specific immunoglobulin E (IgE), eosinophilia, and airway hyperresponsiveness.^2^

Our previous studies found that macrophages expressed B7‐H3 strongly amplified the inflammatory response and augmented proinflammatory cytokines in vitro and in vivo,^3^ which plays an important role in the occurrence and development of asthma, but its complete pathway is still unclear.

MicroRNAs (miRNAs) are key post‐transcriptional modulators that control many cellular pathways and act as critical regulatory elements in multiple immunological settings.^4^ Mature miRNAs repress target gene expression by annealing to the 3‐untranslated region (UTR) of mRNA, resulting in inhibition of mRNA translation and/or increased mRNA degradation.^5^ For Th2 cells, several miRNAs have been implicated in Th2 cell activation and effector differentiation. Such as miR‐155, which is essential for Th2‐mediated inflammation in the lung.^6^

MiR‐29 family includes miR‐29a/b/c. Mature miRNA sequences of miR‐29a/b/c are well conserved, contain identical seed sequences, and are likely to target the same transcripts.^7^ Our previous study found that miR‐29b is low expressed in children with asthma, and it can directly target B7‐H3.^8^ At the same time, through literature review, we found that miR‐29b can target STAT3 at the same time. STAT3 has been reported to be involved in macrophage polarization and may be involved in the immune response of asthma.^9^, ^10^

In this study, we detected the expressions of miR‐29b, B7‐H3 and STAT3 in the peripheral blood mononuclear of children with asthma, and confirmed through in vitro experiments and animal models that miR‐29b participated in the immune inflammatory response of asthma by reducing Th2 cell differentiation through inhibiting B7‐H3 and STAT3. Therefore, miRNA‐29b might be a new therapeutic target for managing asthma.

## 2 MATERIALS AND METHODS

### 2.1 Patients

Hospitalized children from Children's Hospital of Soochow University with asthma exacerbation without treatment were enrolled in this study. The diagnosis of asthma and its severity of asthma exacerbation were confirmed according to Global Initiative for Asthma guidelines (http://ginasthma.org/). Demographic and clinical data of the patients were collected. Peripheral blood samples were collected before treatment with systemic steroids (acute phase) and before discharge (remission phase). The age‐matched controls were chosen randomly from surgery wards without history of lung disease, wheezing, allergy, airway hyperresponsiveness (AHR) and respiratory infections within 4 weeks. The Ethics Committees of Soochow University approved this study, and the parents of all the recruited children provided informed consent.

### 2.2 Isolation of peripheral blood mononuclear cells (PBMCs)

Blood samples were collected in tubes containing ethylene diamine tetraacetic acid (EDTA). Then, blood samples were diluted with phosphate‐buffered saline (PBS, Sigma). PBMCs were isolated using Ficoll‐Paque (Stemcell Technologies) and then isolated PBMCs were washed twice with PBS. The supernatant was discarded, and the pellet was re‐suspended in PBS for flow cytometry and PCR detection.

### 2.3 Real‐time quantitative PCR (qRT‐PCR) for miR‐29b, B7‐H3 and STAT3

According to the manufacturer's protocol, total RNA was extracted from PBMCs using Trizol reagent (Invitrogen). Initially, 10 ng of total RNA was subjected to reverse transcription polymerase chain reaction using the TaqMan MicroRNA Reverse Transcription kit (Invitrogen) according to manufacturer's protocol. qRT‐PCR was performed using TaqMan Universal PCR Master Mix Kit (Invitrogen) in a Bio‐Rad iQ5 Real‐Time PCR System and U6 was used as an endogenous control. The reaction was performed in triplicate according to manufacturer's protocol. After finalization of the qRT‐PCR experiments, the average values of the cycle threshold (Ct) of the reactions in triplicate were determined. Data analysis was performed using the 2^−∆∆Ct^ method. The primer sequences were listed in Table 1.

**TABLE 1:** The primer sequences

Primer	Sequences
B7‐H3	F: CATCCTGAAGCTGACCCAGG
	R: TCCTCACATGGGGGAGGTAG
STAT3	F: TGTCTCATTGCACTGCTGGT
	R: TGGTCCTCATGGTCAGGCTA

### 2.4 Flow cytometry

The cells were adjusted to 10^6^ cells/ml and cultivated with 2 μl cell activation cocktail (BioLegend) for 6 h. And then the cells were washed, resuspended at 1 × 10^6^/ml, and stained with antibodies against CD4 and IL‐4 (BioLegend). After incubation at 4°C for 30 min, the cells were washed twice, and resuspended in PBS, subsequently analyzed on a flow cytometer (BD, FACSVerse).

### 2.5 Macrophage induction in vitro and house dust mite (HDM) stimulation

5 × 10^5^/well THP‐1 cells (Novobio Co. Ltd) were cultured in vitro and PMA (Sigma‐Aldrich) containing (50 ng/ml) medium was used for induction for 6 h, then PMA free complete medium was replaced for further culture for 24 h, THP‐1 cells could be induced into macrophages.

Different doses of HDM (0, 0.02, 0.2, 2, and 20 μg; D. pteronyssinus, Stalledgenes Greer, Lenoir) were used to stimulate macrophages. Cells were collected after 48 h and the expression levels of miR‐29b, B7‐H3 and STAT3 were detected by qRT‐PCR and Western blot. qRT‐PCR detection method was the same as above.

### 2.6 Western blot

Cells were lysed using radio immunoprecipitation assay lysis buffer supplemented with Phenylmethanesulfonyl fluoride. The protein concentration was quantified using a BCA kit and a plate reader (Biosharp). A total of 40 μg protein was subjected to SDS‐PAGE and transferred to polyvinylidene fluoride membrane. Then, the blots were blocked with 5% fat free milk at room temperature for 1 h and incubated overnight at 4°C with the primary antibody. β‐actin mAb (Abcam) was used as the loading control. The primary antibodies used were B7‐H3 (Abcam) and STAT3 (Abcam). Then, the blots were probed with the corresponding HRP‐conjugated secondary antibodies for 1 h at room temperature and visualized using electrochemiluminescence (BIO‐RAD). The band signal intensities were quantified by ImageJ software.

### 2.7 Lentivirus construction and macrophage infection

The expression sequence and interference sequence were designed according to miR‐29b‐3p sequence. The sequence information is as follows (Table 2). The target sequence was synthesized and annealed into double stranded DNA, which was then constructed and linked into a skeleton carrier. Then expression vectors and interference vectors were constructed.

**TABLE 2:** Lentivirus vector sequence

Primer	Sequences
Interference sequence	F: CAACAACACTGATTTCAGATCAATGGTGCTA
	R: CTTGTAGCACCATTGATCTGAAATCAGTGTT
Expression sequence	F:CACCGTAGCACCATTTGAAATCAGTGTTCGAAAACACTGATTTCAAATGGTGCTA
	R:AAAATAGCACCATTTGAAATCAGTGTTTTCGAACACTGATTTCAAATGGTGCTAC

293T cells were co‐transfected with lentiviral vector and packaging plasmid, the virus was packaged. After 48 h, The virus stock was collected, centrifuged over speed, and the concentration was determined. Overexpression (LV527) and interference viruses (LV526) were obtained.

### 2.8 Detection of macrophage function

The induced macrophages were infected with LV526, LV527 and NC viruses to obtain up‐regulated or down‐regulated miR‐29b macrophages. These macrophages were cultured for 24 or 48 h, and then collected for qRT‐PCR and western blot detection. qRT‐PCR detection method was the same as above.

THP‐1 cells or THP‐1 cells with downregulation of miR‐29b were co‐cultured with original T cells. After 24 h, the cells were isolated and the expressions of GATA3 and T‐bet in T cells were detected by qPCR. B7‐H3 or STAT3 antibodies (Abcam) were added into the culture system above, centrifuged after 24 h, the supernatants and cells were collected. The supernatants were detected for IL‐4 and IFN‐γ cytokines (Elabscience), and the cells were detected by flow cytometry.

### 2.9 Luciferase reporter assay

Oligonucleotides corresponding to the miR‐29b binding site in the STAT3‐3′ UTR or a single‐base mutant were synthesized and inserted into the XbaI site immediately downstream from the stop codon of firefly luciferase of the pGL3‐control vector (Novobio Co. Ltd). Human monocyte cell line THP‐1 was obtained from American Type Culture Collection (ATCC, Manassas). THP‐1 cell were co‐transfected in 24‐well plates using Lipofectamine 2000 reagent (Invitrogen) according to the manufacturer's protocol, with 50 ng of the firefly luciferase reporter, 1 ng of the renilla luciferase reporter (Promega) as transfection control, and 100 nM pLenti‐miR‐29b (Novobio Co. Ltd). Firefly and renilla luciferase activities were measured sequentially using dual‐luciferase assays (Promega) 24 h after the transfection and evaluated by the BioTek™ Microplate Reader.

### 2.10 Mice

BALB/c female mice aged 6–8 weeks were raised under specific pathogen‐free conditions and were from Zhao Yan New Drug Research Centre Ltd.

### 2.11 Asthma model and treatment

To induce allergic asthma, mice were intraperitoneally sensitized with 100 μg OVA (Sigma‐Aldrich) and 100 μg LPS (Sigma‐Aldrich) absorbed in 1 ml sterile PBS. After sensitization on days 1 and 8, mice were challenged with 5% OVA inhalation for 30 min every day from day 15 to 21. For miR‐29b treatment, 2 × 10^7^ TU miR‐29b agomiR (NOVOBIO) in 100 µl PBS was administered by intranasal inhalation for seven consecutive days starting from day 15 to day 21. The control group was treated in the same way with PBS. Mice were sacrificed 24 h after the last OVA challenge.

### 2.12 Specimen collection

Twenty‐four hours after the last inhalation of OVA, the mice were sacrificed after anesthesia. Blood was drawing‐out from their hearts and anticoagulated with EDTA. Right lung was ligated and removed for Inflammatory cytokines and histological analysis. The left principal bronchus was cannulated with a polyethylene tube through which the lungs were gently lavaged 3 times with 0.5 ml PBS containing 10% fetal calf serum. A total of 1.5 ml bronchoalveolar lavage fluid (BALF) was collected for Inflammatory cytokines analysis. Western blot was used to detect the expression levels of B7‐H3 and STAT3 in lung tissues, flow cytometry was used to detect the Th cell types in BALF and qRT‐PCR was used to detect the expression levels of IL‐4, IL‐5, IL‐13 and IFN‐γ in lung tissues. The detection method was the same as above. ELISA was used to detect the cytokine levels in lung tissues and BALF. Lung tissues were stained with HE and PAS, and immunohistochemistry was performed.

### 2.13 ELISA

Lung tissue was cut into pieces and grinded on ice after cleaning and weighing. PBS was added into homogenate (Lung tissue: PBS = 1:9). After centrifugation the supernatant was taken out for testing. IL‐4, IL‐5, IL‐13 and IFN‐γ levels in BALF and Lung tissue were determined by commercial ELISA kits (Elabscience) according to the manufacturer's instructions.

### 2.14 Histological analysis of lung sections

The right lungs were first perfused with 4% paraformaldehyde in 0.1 M PBS, fixed with formalin, and embedded with paraffin. Paraffin sections (3 μm) were stained with hematoxylin and eosin (H&E) and periodic acid‐Schiff (PAS) as measurements of eosinophil infiltration and mucus production, respectively. Ten microscopic areas were randomly selected for each mouse. Airway inflammation is scored by eosinophilic cell infiltration under HE staining, which can be divided into 0–4 points. Airway mucus secretion is represented by the percentage of PAS positive epithelial cells under PAS staining. Other paraffin sections were incubated with the rat anti‐B7‐H3 mAb or anti‐STAT3 mAb at 4°C overnight after antigen repair. After incubation with anti‐rat IgG antibody conjugated with horseradish peroxidase (KPL), the specimens were developed using the 3,30‐diaminobenzidine coloration method. 10 sections were randomly selected and average optical density analyzed by using Image‐Pro Plus 6.0 software.

### 2.15 Statistical analysis

The analyses were performed using the Statistical Package for SPSS for windows, version 19.0. The data are presented as mean ± SEM. Student *T*‐test and one‐way ANOVA were performed for the comparisons between groups. Bonferroni's correction method was used for pairwise comparison between groups. Differences between groups were considered statistically significant when *p* < 0.05.

## 3 RESULT

### 3.1 Overexpression of B7‐H3 and Under‐expression of miR‐29b in asthmatic children

A total of 25 children with asthma during acute attack period were enrolled in this study, with the age ranging from 1.66 years old to 14.16 years old. The average age is 5.33 ± 2.85 years old. Among them 15 patients were boys and 10 patients were girls, with the male to female ratio 1.5:1. Peripheral blood was collected before discharge from 23 of the 25 patients as the remission group. The other two children did not collect peripheral blood during remission period due to family refusal. A total of 14 healthy children in the control group were aged from 2.25 years old to 10.16 years old, with an average age of 5.55 ± 2.49 years old. Among them 9 patients were boys and 5 patients were girls, with the male to female ratio 1.8:1. There was no significant difference in age and gender between asthmatic children and control group (*t* = −0.233, *p* = 0.817; *χ* ^2^ = 0.070, *p* = 0.792).

**FIGURE 1:**
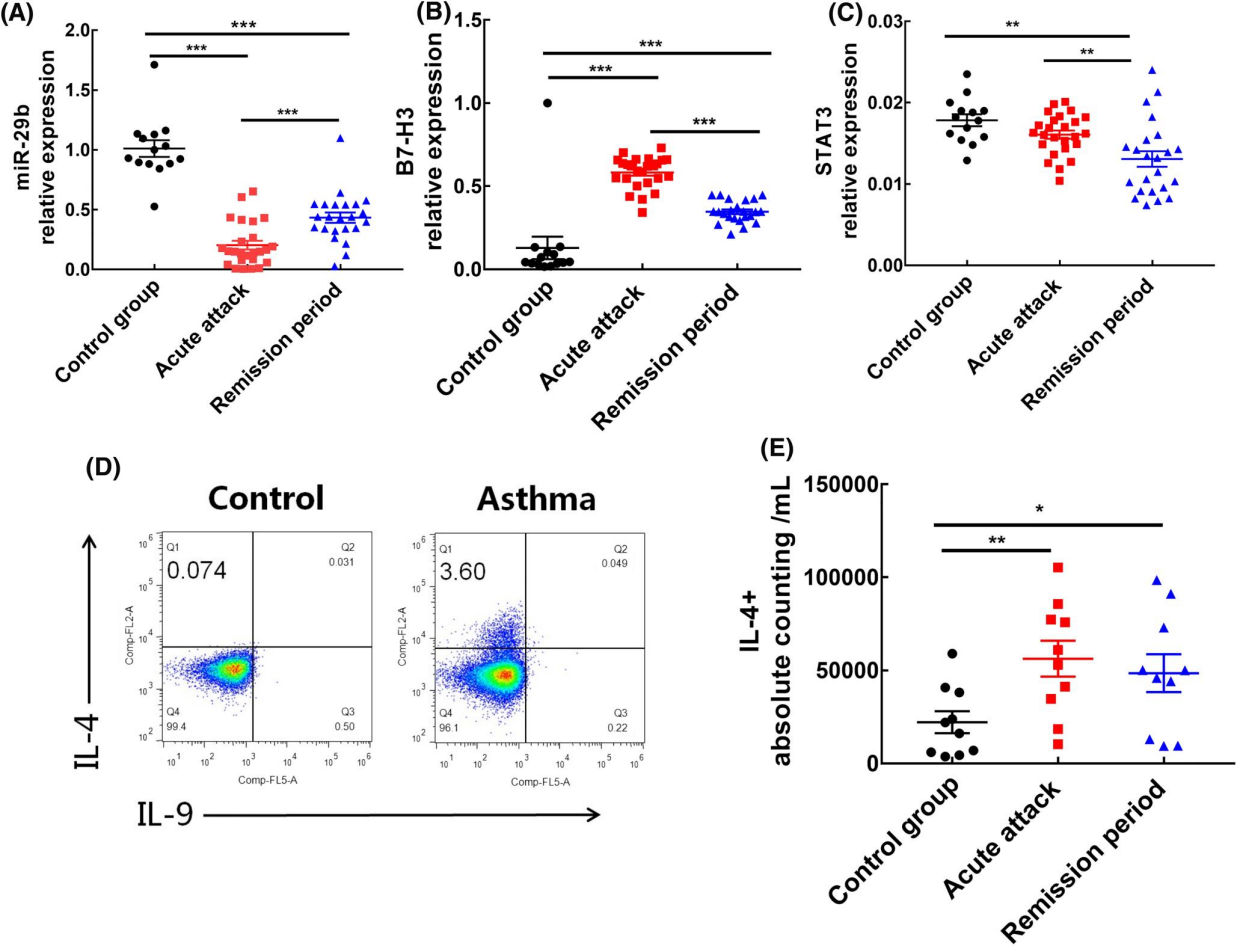
Expression of miR‐29b, B7‐H3 and STAT3 in peripheral blood mononuclear cells and IL‐4 positive cell count of children with asthma. (A–C) The expression levels of miR‐29b, B7‐H3 and STAT3 in peripheral blood peripheral blood mononuclear cells (PBMC) detected by QT‐PCR, respectively. Control group *n* = 14, acute attack group *n* = 25, remission period group *n* = 23. (D,E) T cells detected by flow cytometry. Control group *n* = 10, acute attack group *n* = 10, remission period group *n* = 10. *p < 0.05; **p < 0.01; ***p < 0.0001

We detected the expression of miR‐29b, B7‐H3 and STAT3 in peripheral blood PBMC of asthmatic children (including acute attack period and clinical remission period) and healthy children (control group). Relevant detection data are shown in Figure 1A–C. Compared with healthy children, the expression of miR‐29b was significantly lower in children with acute asthma attack (*p* < 0.001), and was significantly increased after treatment (*p* < 0.001). In contrast, the expression of B7‐H3 was significantly higher in children with acute asthma attacks (*p* < 0.001), and was significantly decreased after treatment (*p* < 0.001). There was no significant difference in the expression of STAT3 between asthmatic children during acute attack period and healthy children (*p* = 0.050). But the expression of STAT3 in asthmatic children during remission period was significantly lower than that in healthy children and children at acute attack period (*p* = 0.001; 0.009).

Flow cytometry showed that the proportion of Th2 cells in asthmatic children was significantly higher than that in healthy children (Figure 1D, 3.600% vs. 0.074%). The absolute count of IL‐4 positive cells in acute attack patients and remission group were significantly higher than healthy children (*p* = 0.007; 0.037). The number of IL‐4 positive cells in children in remission period was lower than that in acute attack period, but with no significantly difference (*p* = 0.584; Figure 1E).

### 3.2 HDM induced macrophages to down‐regulate miR‐29b and overexpress B7‐H3 and STAT3

**FIGURE 2:**
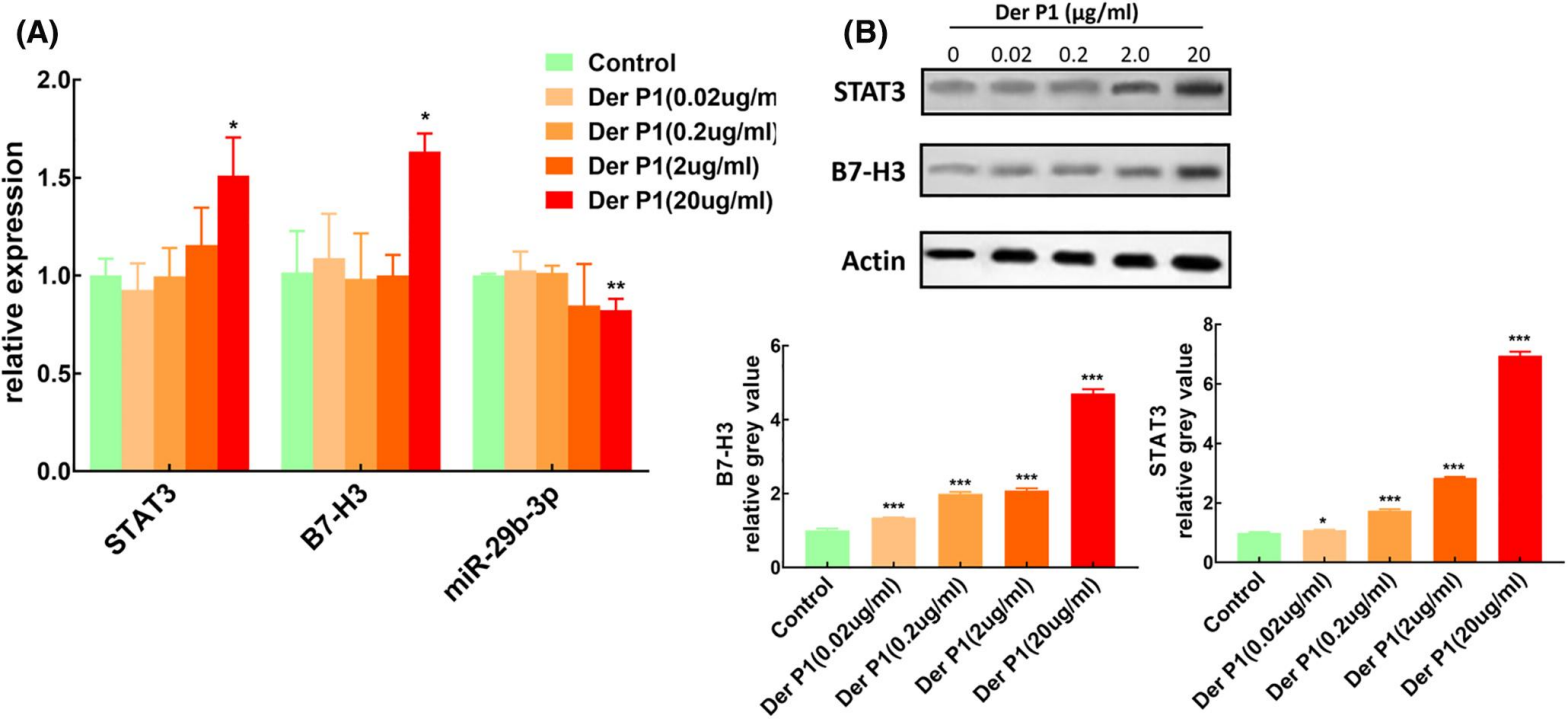
Effects of dust mite stimulation on the expression of miR‐29b, B7‐H3 and STAT3 in macrophages. (A) Detected by QT‐PCR, *n* = 3. (B) Detected by Western blot, *n* = 3. Compared with control group *p < 0.05; **p < 0.01;***p < 0.001

Macrophages were induced and cultured in vitro and stimulated by HDM with different concentrations (Figure 2A). The results showed that the expression level of miR‐29b in macrophages decreased after HDM stimulation, which was concentration‐dependent. High concentration (20 μg/ml) of HDM stimulation could significantly down‐regulate the expression of miR‐29b in macrophages, and the difference was statistically significant compared with the control group (*p* = 0.007). In contrast, the expression level of STAT3 in macrophages increased after HDM stimulation, also with concentration‐dependent. High concentration (20 μg/ml) of HDM stimulation could significantly increase the expression of STAT3 in macrophages, and the difference was statistically significant compared with the control group (*p* = 0.014). Low concentration of HDM stimulation had no obvious effect on the expression of B7‐H3 in macrophages, but high concentration (20 μg/ml) could significantly increase the expression of B7‐H3 in macrophages (*p* = 0.010).

Western blot also confirmed that the protein expression of STAT3 and B7‐H3 in macrophages were increased after HDM stimulation (*p* < 0.001), and this increase was concentration‐dependent (Figure 2B).

### 3.3 Downregulation of miR‐29b induced macrophages to polarize toward M2 type

**FIGURE 3:**
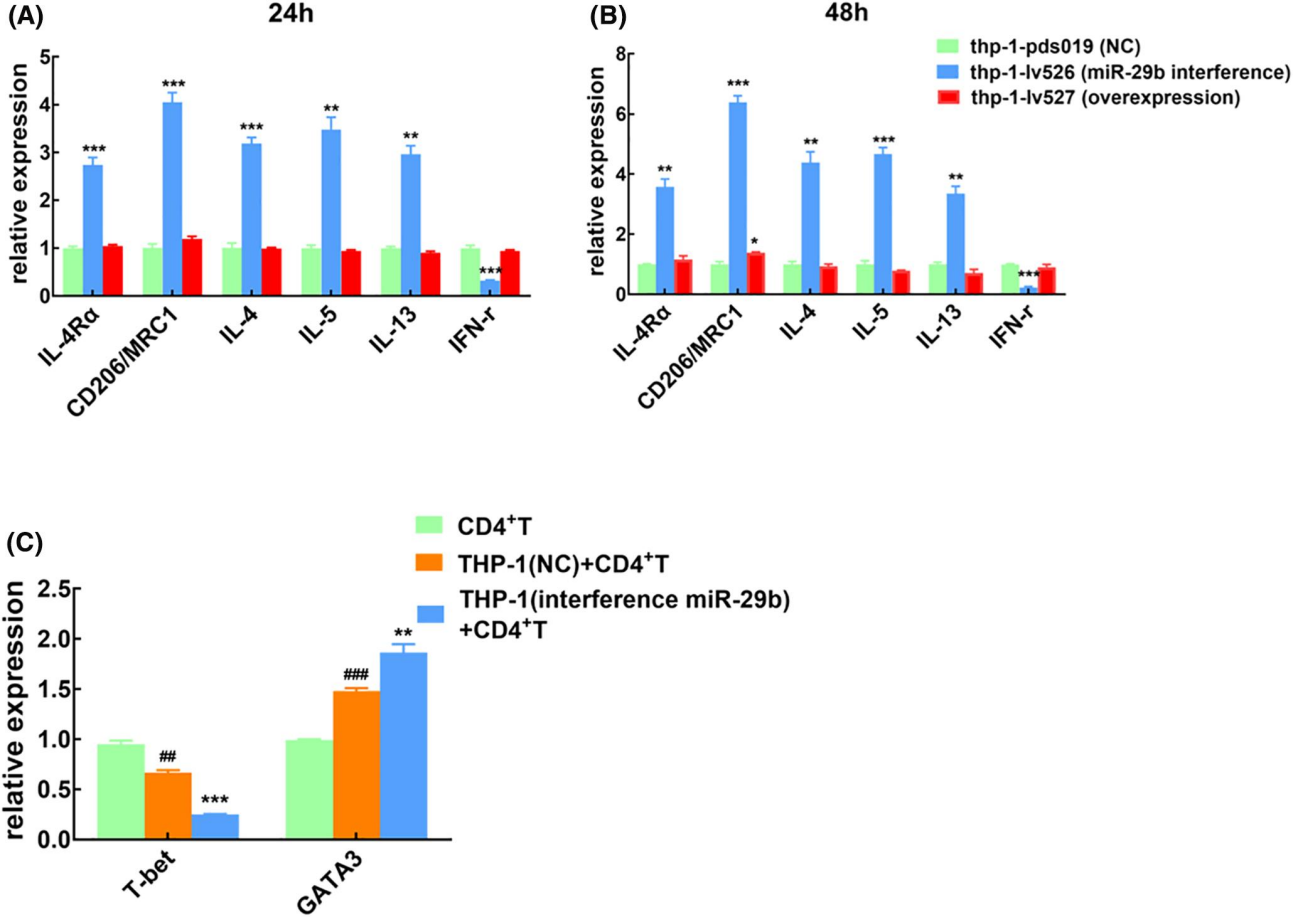
Effect of miR‐29b on macrophage function. (A,B) The polarization of macrophages after up‐regulation or down‐regulation of miR‐29b, detected by QT‐PCR, *n* = 3 (A:24 h, B:48 h). (C) The effect of macrophages on the differentiation of T cells after down‐regulation of miR‐29b, *n* = 3. *p < 0.05; **p < 0.01; ***p < 0.001 compared with NC, normal control group or NC+CD4^+^T group. ##*p* < 0.01; ###*p* < 0.001 compared with CD4^+^T group

In this study, we found that the expression of miR‐29b was low in asthmatic children, and in vitro experiments, the expression of miR‐29b in macrophages was significantly reduced after HDM stimulation. Therefore, we designed an in vitro experiment to regulate the expression of miR‐29b in macrophages and observe its effect on macrophages (Figure 3A,B). It was found that 24 h after down‐regulation of miR‐29b (thp‐1‐lv526), the expression of IL‐4Rα, CD206, IL‐4, IL‐5 and IL‐13 in macrophages was significantly increased, while the expression of IFN‐γ was decreased (*p* < 0.05; Figure 3A). This change is more pronounced in the 48 h (Figure 3B). However, the upregulation of miR‐29b (thp‐1‐lv527) had no significantly effect on the expression of IL‐4Rα, CD206, IL‐4, IL‐5, IL‐13 and IFN‐γ in macrophages.

### 3.4 Downregulation of miR‐29b induced T cells to differentiate into Th2 cells

Compared with single CD4^+^T cell culture, the expression of GATA3 in T cells increased while the expression of T‐bet decreased after adding THP‐1 cells. The difference was statistically significant, suggesting that macrophages can promote T cell differentiation to Th2 type. When we down‐regulated the expression of miR‐29b in THP‐1, the expression of GATA3 in T cells was higher and T‐bet was lower, suggesting the downregulation of miR‐29b induced T cells to differentiate into Th2 cells (Figure 3C).

### 3.5 MiR‐29b directly regulates B7‐H3 and STAT3

**FIGURE 4:**
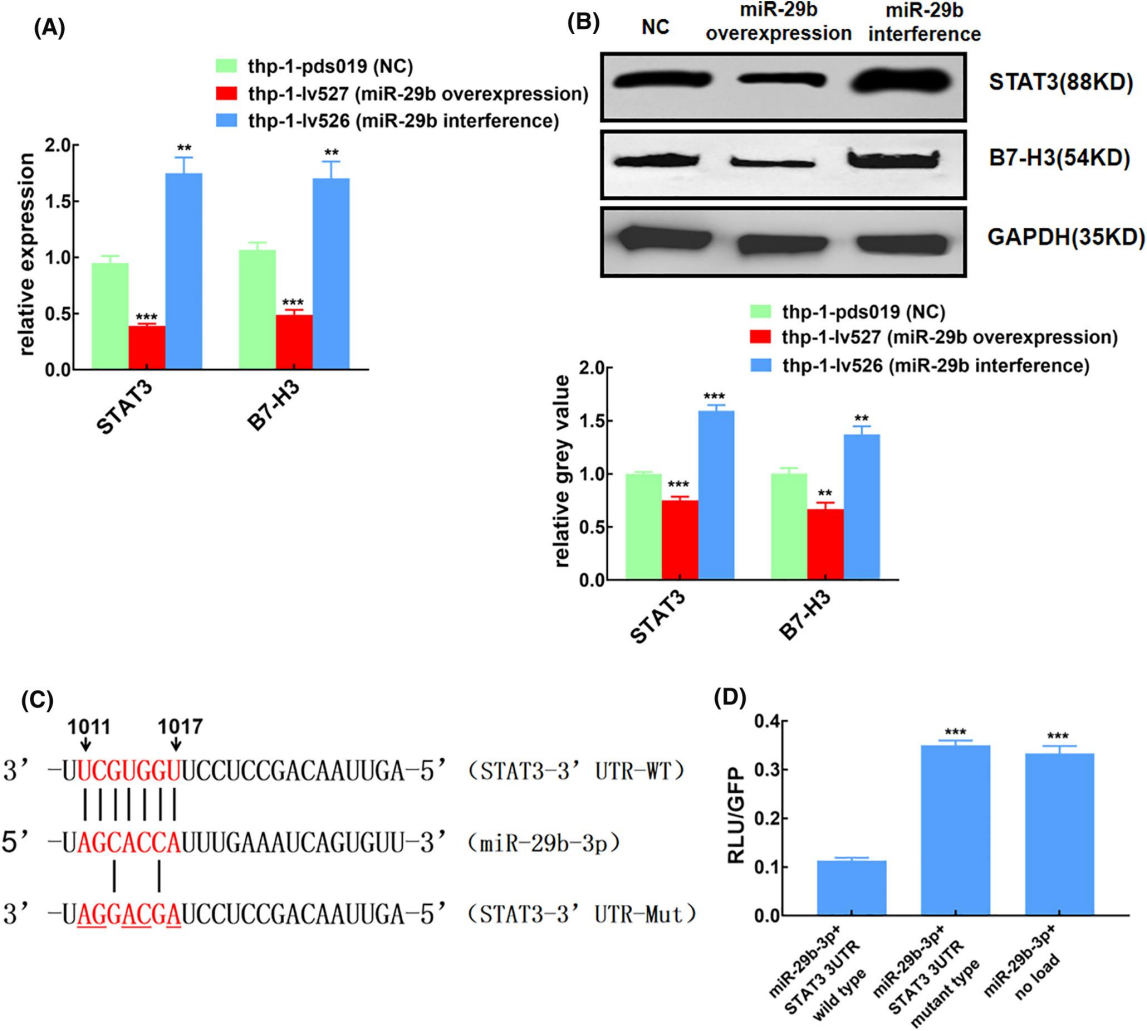
MiR‐29b regulates B7‐H3 and STAT3. STAT3 is the target of miR‐29b confirmed by luciferase reporter assay. (A) Regulation of B7‐H3 and STAT3 expression by miR‐29b on THP‐1 cells, detected by qRT‐PCR, *n* = 3. (B) The expression of B7‐H3 and STAT3 was detected by Western blot, *n* = 3. (C) The binding site of miR‐29b in the 3′ untranslated region (UTR) of the STAT3 mRNA and the mutant sequence of 3′ UTR of STAT3 mRNA. (D) Fluorescein enzyme report. **p < 0.01; ***p < 0.001

In this study, we found that miR‐29b was down‐regulated in asthmatic children, while the expressions of B7‐H3 and STAT3 were increased, and in vitro experiments also confirmed that the expression trend of miR‐29b was opposite to that of B7‐H3 and STAT3. To investigate the possible impact of miR‐29b on STAT3 and B7‐H3, we silence or overexpression the level of miR‐29b in THP‐1 cells and detected the expression levels of B7‐H3 and STAT3 by QT‐PCR and Western blot (Figure 4A,B). Results showed that the expression levels of B7‐H3 and STAT3 in macrophages were significantly increased after the downregulation of miR‐29b (*p* < 0.01). On contrary, the expression levels of B7‐H3 and STAT3 in macrophages were significantly decreased after upregulation of miR‐29b (*p* < 0.01)**.**

Our previous study has confirmed that B7‐H3 is the target gene of miR‐29b.^8^ To examine whether miR‐29b could actually bind to the STAT3 3′‐UTR, we used a luciferase reporter construct with STAT3 3′‐UTR sequence using luciferase technology. The reporter was co‐transfected into THP‐1 cells with miR‐29b and the luciferase activity was measured after 24 h. Transfection of miR‐29b significantly inhibited the luciferase activity of the construct containing the STAT3 3′‐UTR compared to transfection of negative control miRNA or the construct containing the STAT3 3′‐UTR mutant, indicating that miR‐29b directly bind to the STAT3 3′‐UTR (Figure 4C,D).

### 3.6 B7‐H3 and STAT3 promote T cell differentiation to Th2 type

**FIGURE 5:**
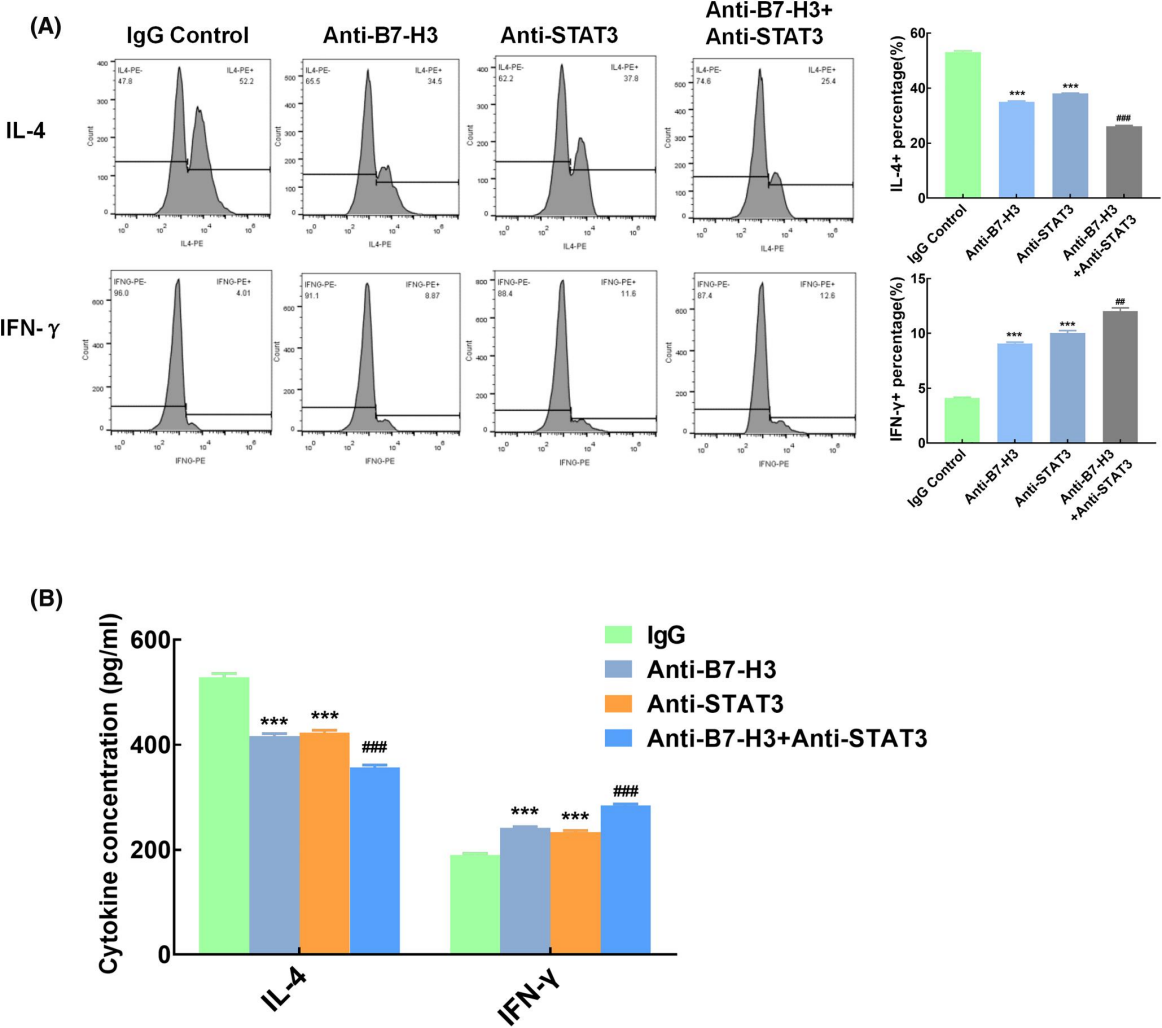
Function of B7‐H3 and STAT3 in regulating Th cell differentiation. CD4^+^T cells was co‐cultured with macrophage (down‐regulated miR29b). (A) CD4^+^T cell differentiation was detected by flow cytometry, *n* = 3. (B) Supernatant cytokines were detected by ELISA, *n* = 3. ***p < 0.001 compared with IgG control group; ##*p* < 0.01 compared with anti‐B7‐H3 group and anti‐STAT3 group

To investigate the role of B7‐H3 and STAT3 in the pathogenesis of asthma and whether there is synergistic effect between them. Macrophages (miR‐29b down‐regulated) were co‐cultured with CD4^+^T cells. IgG control, B7‐H3 antibody, STAT3 antibody or both B7‐H3 and STAT3 antibody were added into the system. T cell surface markers were detected by flow cytometry and supernatant cytokines were detected by ELISA after 24 h of culture (Figure 5).

The results showed that adding B7‐H3 antibody or STAT3 antibody both reduced the percentage of IL‐4^+^T cells and increased the percentage of IFN‐γ^+^T cells, which had a significant difference compared with the IgG group (*p* < 0.001). However, adding both B7‐H3 and STAT3 antibody, the percentage of IL‐4^+^T cells was significantly lower as well as the percentage of IFN‐γ^+^T cells significantly higher compared with adding B7‐H3 of STAT3 antibody alone (*p* < 0.01; Figure 5A).

The cytokines in the supernatant also showed the same trend (Figure 5B). Compared with the IgG control group, the level of IL‐4 in the supernatant was significantly decreased after adding B7‐H3 or STAT3 antibody, while the level of IFN‐γ was increased. After adding both B7‐H3 and STAT3 antibody, the level of IL‐4 in supernatant was lower, while the level of IFN‐γ was higher (*p* < 0.001).

These results indicated that T cells differentiated to Th1 cells after blocking B7‐H3 or STAT3, which proved that B7‐H3 and STAT3 may promote T cells differentiated to Th2 cells.

### 3.7 MiR‐29b alleviates asthma inflammation by inhibiting B7‐H3 and STAT3 in vivo

To further clarify the mechanism of miR‐29b in vivo, we established mice asthma model and specifically upregulated miR‐29b in lungs by aerosol gene transfection. Many asthmatic syndromes were observed in normal control group with asthma, such as cough, tachypnea, dyspnea. And in the miR‐29b group asthma‐like symptoms were relieved, only mild wheezing and tachypnea.

**FIGURE 6:**
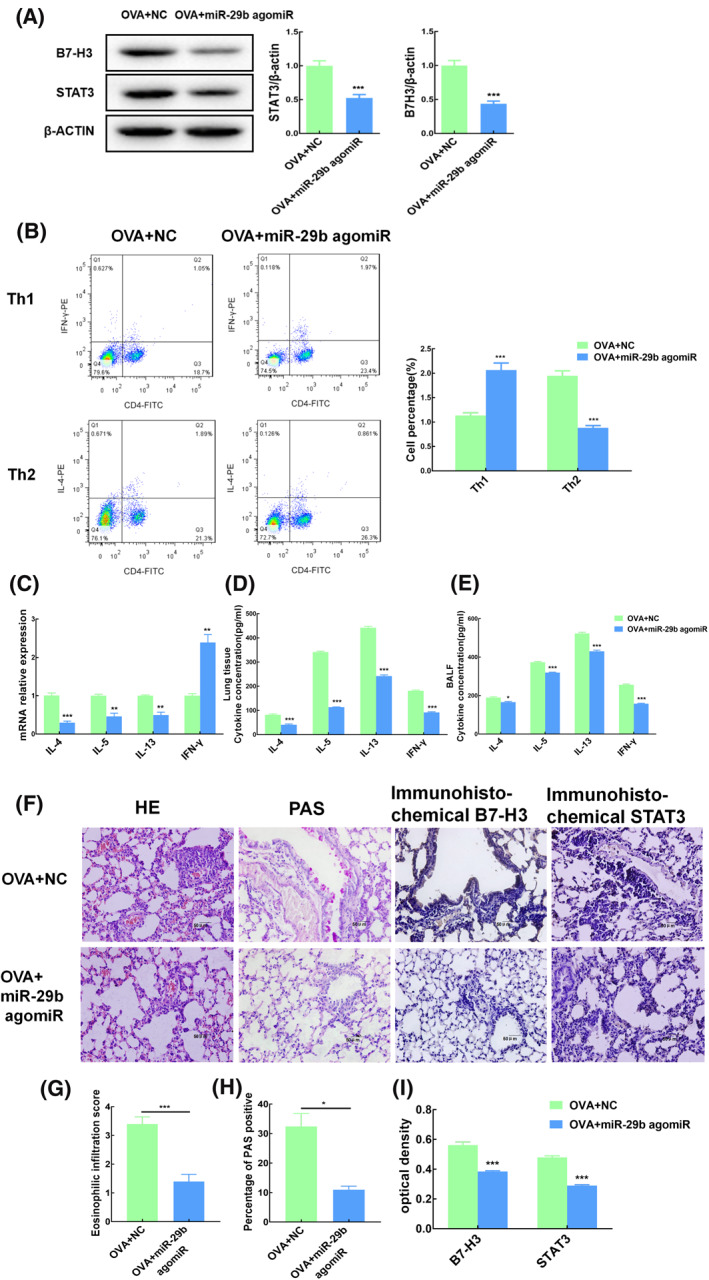
MiR‐29b inhibits asthma inflammation. Mice asthma model was made by OVA sensitization and stimulation. MiR‐29b agomiR specifically up‐regulated miR‐29b in lung through aerosol gene transfection system (*n* = 3). (A) The expression levels of B7‐H3 and STAT3 in lung tissues of mice were detected by Western blot. (B) The classification of Th cells in bronchoalveolar lavage fluid (BALF) was detected by flow cytometry. (C) The expression of inflammatory factors in lung tissue was detected by qRT‐PCR. (D) Cytokine concentration in lung tissue was detected by ELISA. (E) Cytokine concentration in BALF was detected by ELISA. (F) HE staining, periodic acid‐Schiff (PAS) staining and immunohistochemistry of lung tissue (original magnification ×400, scale bars = 50 μm). (G) HE staining, airway inflammation was indicated by eosinophilic infiltration score. (H) PAS staining, the airway mucus level was expressed as PAS positive percentage, (I) the optical density of immunohistochemistry. NC, normal control. **p < 0.01 compared with normal control group; ***p < 0.001 compared with normal control group

After the up‐regulation of miR‐29b, the expression levels of B7‐H3 and STAT3 in lung were significantly decreased (*p* < 0.001; Figure 6A). Flow cytometry showed an increased proportion of Th1 cells and a decreased proportion of Th2 cells in lung tissue (*p* < 0.001; Figure 6B). PCR showed that the expression of IL‐4, IL‐5 and IL‐13 was decreased while the expression of IFN‐γ was significantly increased in lung tissues (*p* < 0.01; Figure 6C). The levels of cytokines in lung tissues and BALF detected by ELISA also showed that the levels of IL‐4, IL‐5 and IL‐13 were increased, while the levels of IFN‐γ were decreased (*p* < 0.05; Figure 6D,E).

As shown in Figure 6F, up‐regulation of miR‐29b significantly alleviated airway stenosis and mucosal epithelial hyperplasia, reducing inflammatory cell and eosinophil infiltration in lung tissues. There were significant differences in airway inflammation scores between the two groups (*t* = 5.774, *p* < 0.001; Figure 6G). PAS staining indicated that the up‐regulation of miR‐29b significantly reduced airway mucus secretion in asthmatic mice (*t* = 4.737, *p* = 0.013; Figure 6H). Immunohistochemistry also confirmed that the expression levels of B7‐H3 and STAT3 in lung tissues were decreased after up‐regulation of miR‐29b (Figure 6F,I).

## 4 DISCUSSION

Asthma is characterized by chronic inflammation of the airways, contraction of smooth muscle, overproduction of mucus, and remodeling of the airway wall. In many forms of asthma, there is an accumulation of eosinophils, which is controlled by type Th2 lymphocytes of the adaptive immune system. This make IL‐4, IL‐5 and IL‐13 in response to the presentation of an allergen by antigen extractor in the airway.^11^

Professional antigen presenting cells include dendritic cells, monocytes‐macrophages, and B lymphocytes. Macrophages are one of the main cells in the lung, and the inhaled allergens are first recognized by macrophages. Studies showed that macrophages play an important role in the immune mechanism of asthma.^12^ Therefore, macrophage was selected as the research object in this study.

The properties of macrophages are determined by the expression of surface signaling molecules and their own polarization.^13^ Our previous study confirmed that B7‐H3 expressed by macrophages can participate in the immune process of asthma.^3^, ^14^ In this study, we confirmed that the expression of B7‐H3 in children with asthma was increased again, and the expression of B7‐H3 in macrophages was significantly increased after the stimulation of HDM in vitro. After the addition of B7‐H3 antibody in the co‐culture system of macrophages and naive T cells, the differentiation of Th2 cells decreased and the secretion of IL‐4 also decreased, which reconfirmed that B7‐H3 participated in the occurrence and development of asthma by regulating the differentiation of Th cells.

Polarization is another aspect of the properties of macrophages, which is closely related to the immunopathology of asthma.^15^, ^16^ According to different microenvironment, macrophages can differentiate into different subtypes, mainly divided into two types: classical activated macrophages (M1 type) and selectively activated macrophages (M2 type).^17^ Among them, M2 macrophages are activated by IL‐4 and IL‐13, which play a role in enhancing Th2 inflammatory response, and participating in angiogenesis and tissue repair.^18^ Studies have shown that the number of M2 type macrophages in the BALF of patients with asthma is significantly increased, which is related to the severity of asthma.^19^ The mouse model of asthma induced by OVA also shows that the number of M2 type macrophages is significantly increased.^16^ There are many signaling molecules and pathways involved in macrophage polarization, such as JNK, PI3K/Akt, Notch, Jak/STAT, TGF‐β/Smad and TLR/NF‐κB. STAT3 signal transduction plays an important role in macrophage polarization. It has been reported in tumor diseases and endocrine diseases that the JAK2/STAT3 pathway can induce the macrophages transformed to M2 type.^9^, ^10^ In this study, we found that the expression of STAT3 in peripheral blood of patients with asthma was increased. Vitro experiments showed that HDM stimulation could increase the expression of STAT3 in macrophages. After the addition of STAT3 antibody in the co‐culture system of macrophages and naive T cells, the differentiation of Th2 cells decreased and the secretion of IL‐4 also decreased, which indicated that STAT3 may also involved in the immune mechanism of asthma.

The properties of cell cannot be separated from the regulation of its own genes. MiRNA plays an important role in the regulation of cell proliferation, differentiation, metabolism and apoptosis, and participates in the immune regulation process of immune cells.^20^ Our previous study found that miR‐29b was a miRNA differentially expressed in asthmatic patients, and can bind specifically to B7‐H3.^8^ Previous studies have confirmed that miR‐29b could reverses the imbalance of Th1/Th2 responses and decreases eosinophils recruitment in the airway, deducing miR‐29b might be an attractive candidate target for asthma treatment.^21^ In this study, we found that the expression of miR‐29b was decreased in peripheral blood mononuclear cell of children with asthma, and the expression of miR‐29b was decreased after HDM stimulation in vitro. Vitro experiments showed that down‐regulation of miR‐29b expression in macrophages could induce T cells to differentiate into Th2 type. Animal models also confirmed that the upregulation of miR‐29b can reduce the immune inflammatory response of asthma. These all confirmed that miR‐29b was indeed involved in the immune response of asthma. Moreover, we confirmed that STAT3 is also the target gene of miR‐29b by fluorescence enzyme assay. Vitro experiments also confirmed that the expression of B7‐H3 and STAT3 in macrophages decreased after the up‐regulation of miR‐29b, whereas the expression of B7‐H3 and STAT3 in macrophages increased after the down‐regulation of miR‐29b. Therefore, we speculated whether miR‐29b could act on both B7‐H3 and STAT3, not only affecting the surface signal molecules of macrophages, but also affecting the polarization of it, so as to strengthen the regulation of downstream T cells.

To confirm whether both pathways play a role in the pathogenesis of asthma, we co‐cultured THP‐1 cells (which is down‐regulated miR‐29b) and naive T cells, and blocked one of the pathways respectively. STAT3 is an intracellular molecule. But when STAT3 does its job, part of STAT3 can be recruited to the cell membrane. Studies showed that recruitment of STAT3 to the plasma membrane is essential for its phosphorylation.^22^ S‐palmitoylation can target proteins to membranes, and STAT3 needs to be recruited to the plasma membrane to interact with JAK2.^23^ So we use STAT3 antibodies that can bind to STAT3 on the cell membrane and block it. We found that blocking B7‐H3 or STAT3 respectively could prevent T cells from differentiating into Th2 type, and blocking both pathways at the same time had a more significant effect. It is confirmed that miR‐29b is indeed involved in the pathological process of asthma through two pathways.

**FIGURE 7:**
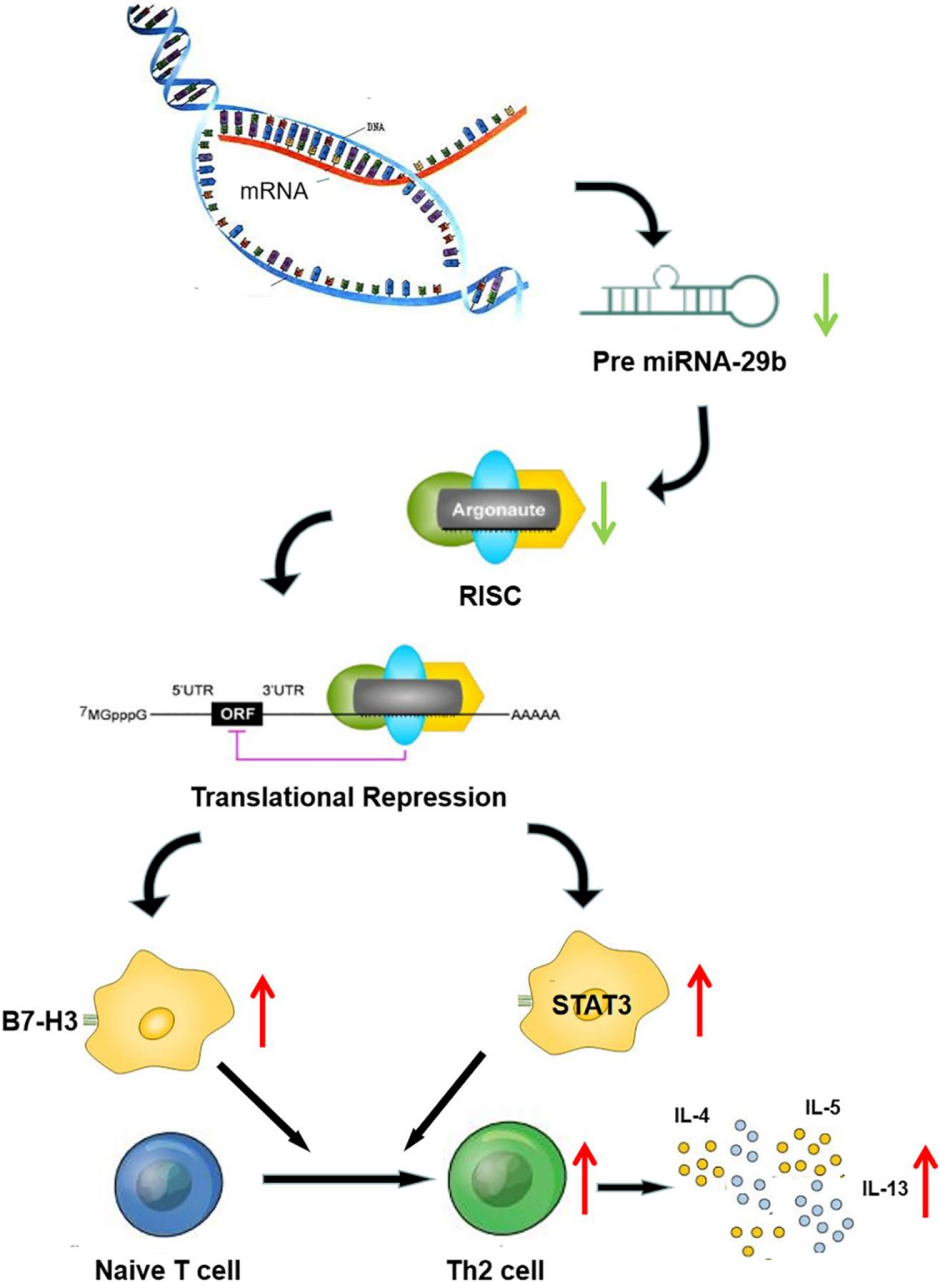
Schematic diagram of the effect of miR‐29b on immune inflammatory response of asthma by regulating B7‐H3 and STAT3

Therefore, we concluded that the down‐regulation of miR‐29b in asthmatic patients reduced the inhibition of the expressions of B7‐H3 and STAT3. This, on one hand resulted in the increased expression of B7‐H3 on the surface of macrophages, and on the other hand resulted in the increased expression of STAT3 in macrophages. The upgraded STAT3 resulted in the polarization of macrophages to M2‐type. Together, these two pathways lead to differentiation of naive T cells into Th2 cells, releasing a large number of inflammatory factors (IL‐4, IL‐5 and IL‐13) and aggravating the progression of asthma. The specific schematic diagram is showed in Figure 7. The results of this study may provide new ideas and potential intervention targets for the treatment of asthma.

## CONSENT FOR PUBLICATION

Not applicable.

## CONFLICT OF INTERESTS

The authors declare that they have no competing interests.

## AUTHOR CONTRIBUTIONS

Designed the study: Zhengrong Chen and Chuangli Hao. Provided the statistical analysis: Yanyu He and Weili Zhang. Collected clinical sample: Xinxing Zhang. Animal model establishment: Gang Li. Drafted the initial manuscript: Wenjing Gu and Li Huang. Revised the manuscript: Yongdong Yan and Wei Ji. All authors approved the final content of this manuscript.
